# The association between smoking and COVID-19 symptoms, severity, and post-COVID-19 symptoms: a cross-sectional survey study

**DOI:** 10.3389/fpubh.2026.1746370

**Published:** 2026-07-06

**Authors:** Musaad A. Alshammari, Fawaz Alasmari, Aleksandra Maria Rogowska, Sary Alsanea, Haneen Alotaibi, Reem Almuneef, Tahani K. Alshammari

**Affiliations:** 1 Department of Pharmacology and Toxicology, College of Pharmacy, King Saud University, Riyadh, Saudi Arabia; 2 Institute of Psychology, Faculty of Social Sciences, University of Opole, Opole, Poland; 3 King Saud University, Riyadh, Saudi Arabia

**Keywords:** cigarette smoking, conventional cigarette, coronavirus, COVID-19, E-Cig, electronic cigarette

## Abstract

**Introduction:**

Coronavirus disease 2019 (COVID-19) is globally linked to serious health threats. Understanding the risk factors associated with COVID-19 would improve our health status and preparedness for pandemics. Smoking is linked to adverse and long-term health consequences.

**Methods:**

The central goal is to examine the factors associated with COVID-19 severity, including smoking E-Cigs and conventional cigarettes, using the Cigarette Dependence Index (a validated questionnaire), and considering sociodemographic variables and disease severity. The survey was distributed online from 19 January to 1 March, 2023; of 560 responses, 262 were included. We found that most cases in the fourth infection exhibited a higher percentage of symptom-free individuals and shorter disease duration.

**Results:**

We found that most cases in the fourth infection exhibited a higher per-centage of symptom-free individuals and shorter disease duration. The logistic regression analysis showed that singles were four times more susceptible than married individuals, and males were two times more likely to present coronavirus symptoms than females. The binomial logistic regression studies showed that post-coronavirus symptoms were higher in men and individuals with asthma.

**Conclusion:**

This evidence supports promoting innovative solutions to prevent smoking and establishing a sustainable emergency response system based on the COVID-19 experience.

## 1 Introduction

The Coronavirus disease 2019 (COVID-19) pandemic has impacted global health, the economy, and social lives. Although the global threat of the COVID-19 pandemic has been downgraded, some of its associated changes have been retained. For example, some aspects of societal norms have been shifted, including factors related to disease protection and cultural tightness (1). As a consequence, research norms have been changed. Yet the need to employ diverse study approaches and research questions is paramount (2). Among previous pandemics, the lessons learned from COVID-19 were significant and deeply rooted (3).

Notably, a previous review comprehensively examined studies of COVID-19’s impacts on environmental sustainability. It reported a lack of multiple sustainability-relevant domains, including the identification of human well-being within a dynamic analytical framework. Furthermore, complex interactive and adaptive behaviors have emerged (4). For instance, pandemic-related risk adaptation behavior, including smoking, has developed (5). The elevation in the smoking rate has been recognized before the COVID-19 pandemic, particularly in high-income societies (6). Reports on sustainable development research indicated that good health and well-being, a domain of the Sustainable Development Goals (SDGs), has received positive consideration for rebuilding and strengthening its foundations (7).

It is well acknowledged that the majority of E-Cig users are the younger population, and the rate of conversion from traditional smoking to E-Cig is increasing significantly (8). Accumulating evidence indicates that increased use of E-Cigs can irritate lung cells and tissues (9, 10), which is linked to inflammation, hyperresponsiveness, and infectious diseases (11, 12). In a preclinical setting, exposure to E-Cig prenatal stages is linked to behavioral changes at adolescence, including elevated risk of tobacco addiction, abnormal rodent behavior, and decreased cognitive capacity. Highlighting potential long-term consequences of electronic cigarette consumption during pregnancy (13). A recent study indicated the unmet need to examine smoking behavior (14).

Available evidence indicates an association between nicotine use and the risk of COVID-19 (15–17). Smoking by itself is connected to deteriorated respiratory function. A meta-analysis study found that smoking increases the risk of acquiring severe COVID-19 infection by about twofold (18). In the early waves of the COVID-19 pandemic, using a retrospective setting, we have reported that the severity of COVID-19 outcomes is linked to smoking (19). Smoking prevails in more than 20% of the Saudi population (20).

Therefore, studies regarding the COVID-19-smoking association, including smoking behavior and the severity of infectious disease symptoms, are needed. Recent studies suggest that E-Cig use may cause dependence by acting on nicotinic receptors or neurotransmitters (21, 22), potentially increasing the risk of chronic E-Cig use and affecting various body systems, including the respiratory system, which is a target of COVID-19.

Here, we aim to assess a potential relationship between smoking and COVID-19 in the adult population currently residing in Saudi Arabia (KSA). In this work, we considered multiple socioeconomic factors, such as age (23), gender (24), and marital status (25), that have been investigated in the context of COVID-19 symptom severity. Profiling the smoking and COVID-19 association is important to understand gaps in our status and help rebuild our community after the pandemic, and face future pandemics.

## 2 Materials and methods

### 2.1 Study design

A cross-sectional study, survey-based.

Inclusion criteria

Smoker and non-smoker adult subjects (18 years or older) diagnosed with COVID-19 and dependence on smoking (E-Cig or conventional cigarette) were included. The participants were asked several questions to confirm whether the subject was dependent on smoking. These questions follow the Diagnostic and Statistical Manual of the American Psychiatric Association (DSM-IV) criteria for the diagnosis of tobacco use disorder.^1^

Exclusion criteria

COVID-19 negative, and if the subject refuses to consent or the patient cannot consent. People under 18 years of age or not residing in Saudi Arabia were not eligible to participate.

Study duration

The questionnaire was disseminated from 19 January to 1 March, 2023.

### 2.2 Distribution, recruitment and data collection

We used email and social media to promote our survey participation, which is a low-cost strategy. An email and a social media advertising campaign are designed to recruit participants for the online survey. The survey is published on different platforms such as Facebook, Instagram, WhatsApp, and Twitter. We offer incentives in various forms, including gift cards and discounts, to facilitate recruitment. The questionnaire was adapted from an original web-based survey informed by the literature, and validated scales were used to collect data on demographic information, smoking history, comorbidities, and COVID-19 severity among the adult population residing in Saudi Arabia (KSA). The questionnaire contained demographic information, including the smoking index survey (modified version of E-cigarette/conventional cigarettes), comorbidity, and COVID-19 status (Supplementary material). Because answers to all questions were mandatory, and participants could not proceed to the next question unless they answered all of the questions in a given section, there was no missing data in the database. The survey was distributed in both English and Arabic. We translated it into Arabic, consulted five experts, and conducted a pilot study. The survey included multiple questions representing different aspects and was distributed via REDCap (Vanderbilt University, USA). Survey items include:

1. Demographic information.
2. History and type of conventional smoking and E-Cig.
3. Smoking frequency (Smoking Dependency).
4. Comorbidity.
5. COVID-19 Severity.
6. Knowledge of COVID-19 prevention measures.

### 2.3 Study variables

To account for the impact of these factors in the analysis, the following variables were considered: patients’ age, gender, state of residence, pregnancy status, smoking history, smoking type, nicotine percentage, the presence of comorbid conditions, and COVID-19 disease severity. Independent Variables include adverse effects associated with E-Cig and cigarette use, anxiety and depression symptoms, and hospitalization time. The estimated completion time for the survey is 10–15 min.

### 2.4 Ethical considerations

The studies were reviewed and approved by the Institutional Review Board IRB at King Saud University. The study complied with local guidelines and the institutional IRB instructions with an ethics reference number (E-22-7267).

### 2.5 Statistical analysis

Frequencies in most variables were calculated as the number of participants and the percentage in each category. Contingency tables and Pearson’s χ^2^ test of independence were used to assess the association between current smoking and other demographic and health-related variables. The effect size was assessed using the *φ* statistic.

The study used logistic regression to examine the relationships among COVID-19 symptoms, demographics, comorbidities, and smoking habits. Predictor variables included in the regression models were selected based on prior evidence and theoretical relevance to COVID-19 symptom severity and smoking behavior. Demographic factors such as age, gender, relationship status, and occupational status were included due to their known associations with disease outcomes and health behaviors. Pre-existing health conditions (disability, asthma, diabetes, heart disease, mental disorder, neurological disorder, immunological disease) were incorporated as important comorbidities influencing COVID-19 severity. Smoking-related variables were chosen to represent different aspects of tobacco use and dependence, including current smoking status, smoking type, number of cigarette brands used, difficulty refraining from smoking, morning smoking habits, and nervousness when smoking is prohibited. Variables with low variability, high collinearity, or limited relevance were excluded to ensure model stability and interpretability. First, the composite scores were calculated as a sum of infections during the pandemic for two explained variables: (1) the number of coronavirus symptoms and (2) the severity of COVID-19 symptoms. Higher scores indicate a greater number of symptoms and more severe symptoms of COVID-19 across all four infection times. Ordinal regression models were applied for these two outcome variables, and a set of potential predictors, including demographic variables (age, gender, relationship status, and occupational status), pre-existing diseases (disability, asthma, diabetes, heart disease, mental disorder, neurological disorder, and immunological disease), and variables related to smoking (current smoking status, types of smoking, number of smoking cigarette brands, difficulties to not smoking, morning smoking, and nervousness if smoking is prohibited). Also, binomial logistic regression was performed for post-coronavirus status (coded as symptoms of post-COVID = 1, no symptoms = 0) and the same set of predictor variables as in the previous ordinal regression models.

In logistic regression, the Adjusted Odds Ratio (AOR) represents the odds of an outcome occurring in one group compared to another, after controlling for other variables in the model. For interpreting the magnitude of a positive association: AOR from 1.1 to 1.5 is considered as weak (small), 1.6 to 3.0 as moderate, and AOR > 3.0 is large; whereas for negative associations, the effect size is interpreted as follows: AOR between 0.67 and 0.90 as weak, 0.34 to 0.66 as moderate, and AOR < 3.33 as large (26). All statistics were calculated using JAMOVI ver. 2.2.5 for Windows.

## 3 Results

### 3.1 Participant characteristics

Among the participants, most were males aged 18–30, single, students, of Saudi nationality, and living in the Central Region (Table 1). Most respondents suffered from no long-standing disease, disability, or asthma. Several people are declared to suffer from other conditions, including diabetes, heart disease, mental disorders, immunological diseases, and neurological disorders.

**Table 1:** Demographic characteristics of participants (*N* = 238).

Variable	Categories	*n*	%
Age	Younger than 18	9	3.78
	18–30	144	60.5
	30–45	70	29.41
	Older than 45	15	6.3
Gender	Female	113	47.48
	Male	115	48.32
Relationship status	Single	142	59.66
	Married	16	6.72
	Widow/er	78	32.77
Occupation status	Student	119	50
	Housewife	9	3.78
	Employee	101	42.44
	Other	3	1.26
Nationality	Non-Saudi	26	10.92
	Saudi	210	88.24
Geographic region	The Central Region	169	71.01
	The Eastern Region	2	0.84
	The Western Region	47	19.75
	The Northern Region	10	4.20
	The Southern Region	10	4.20
Chronic diseases	Disability	60	25.21
	Asthma	25	10.50
	Diabetes	12	5.04
	Heart disease	12	5.04
	Mental disorder	12	5.04
	Neurological disorder	3	1.26
	Immunological disease	7	2.94
	Other	15	6.30
	No long-standing	172	72.27

### 3.2 Experience COVID-19 among participants

All respondents (*N* = 238) suffered from the coronavirus. Only 8 people were vaccinated during the pandemic (3.36%), of whom 3 received 2 doses (1.26%), and 5 received 3 doses (2.10%). Positive test results for COVID-19 were confirmed retrospectively one time in 183 participants (76.89%), two times in 41 people (17.23%), three times in 7 individuals (2.94%), and four times in 5 people (2.10%). In the sample, 87 people (36.56%) reported post-coronavirus symptoms. Table 2 shows the frequencies of COVID-19 symptoms, including symptom types, subjective assessment of symptom severity, symptom duration, and hospitalization related to COVID-19. The number of symptoms was summarized from the first to fourth infections reported for each participant to calculate a composite score of symptom count during the pandemic (ranging from 0 to 21; M = 4.43, SD = 2.92, Mdn = 4, *N* = 238). Summarizing the severity of symptoms across all four time points during the pandemic, the composite score for individuals ranges from 0 to 8, with an average of 2 (M = 2.31, SD = 1.35, Mdn = 2, *N* = 238).

**Table 2:** Characteristics of COVID-19 infections during the pandemic.

Variable	Categories	1st infection		2nd infection		3rd infection		4th infection	
		*n*	%	*n*	%	*n*	%	*n*	%
COVID-19 symptoms	No Symptoms	10	4.20	191	80.25	229	96.22	234	98.32
	Fever	74	31.09	11	4.62	2	0.84	1	0.42
	Cough	165	69.33	36	15.13	8	3.36	4	1.68
	Tiredness	149	62.61	29	12.19	6	2.52	2	0.84
	Loss of taste or smell	167	70.17	23	9.66	5	2.10	2	0.84
	Difficulty breathing	106	44.54	12	5.04	4	1.68	1	0.42
	Muscle aches	113	47.48	23	9.66	5	2.10	2	0.84
	Other	88	36.98	11	4.62	4	1.68	1	0.42
Severity of COVID-19	No symptoms	11	4.62	189	79.41	229	96.22	234	98.32
	Mild	56	23.53	23	9.66	7	2.94	3	1.26
	Moderate	118	49.58	18	7.56	2	0.84	0	0.00
	Severe	53	22.27	8	3.36	0	0.00	1	0.42
Duration of COVID-19 symptoms	Any 1 day	182	76.47	189	79.41	229	96.22	234	98.32
	5 days or less	4	1.68	0	0.00	0	0.00	0	0.00
	6 days to 2 weeks	46	19.33	44	18.49	8	3.36	4	1.68
	2 weeks to 1 month	0	0.00	0	0.00	0	0.00	0	0.00
	More than a month	6	2.52	5	2.10	1	0.42	0	0.00
Hospitalization	Not hospitalized	205	86.13	234	98.32	234	98.32	235	98.74
	Less than 2 days	5	2.10	0	0.00	0	0.00	0	0.00
	2–5 days	13	5.46	1	0.42	2	0.84	1	0.42
	5–9 days	10	4.20	1	0.42	1	0.42	1	0.42
	More than 9 days	5	2.10	2	0.84	1	0.42	1	0.42

### 3.3 Smoking behavior

**Figure 1:**
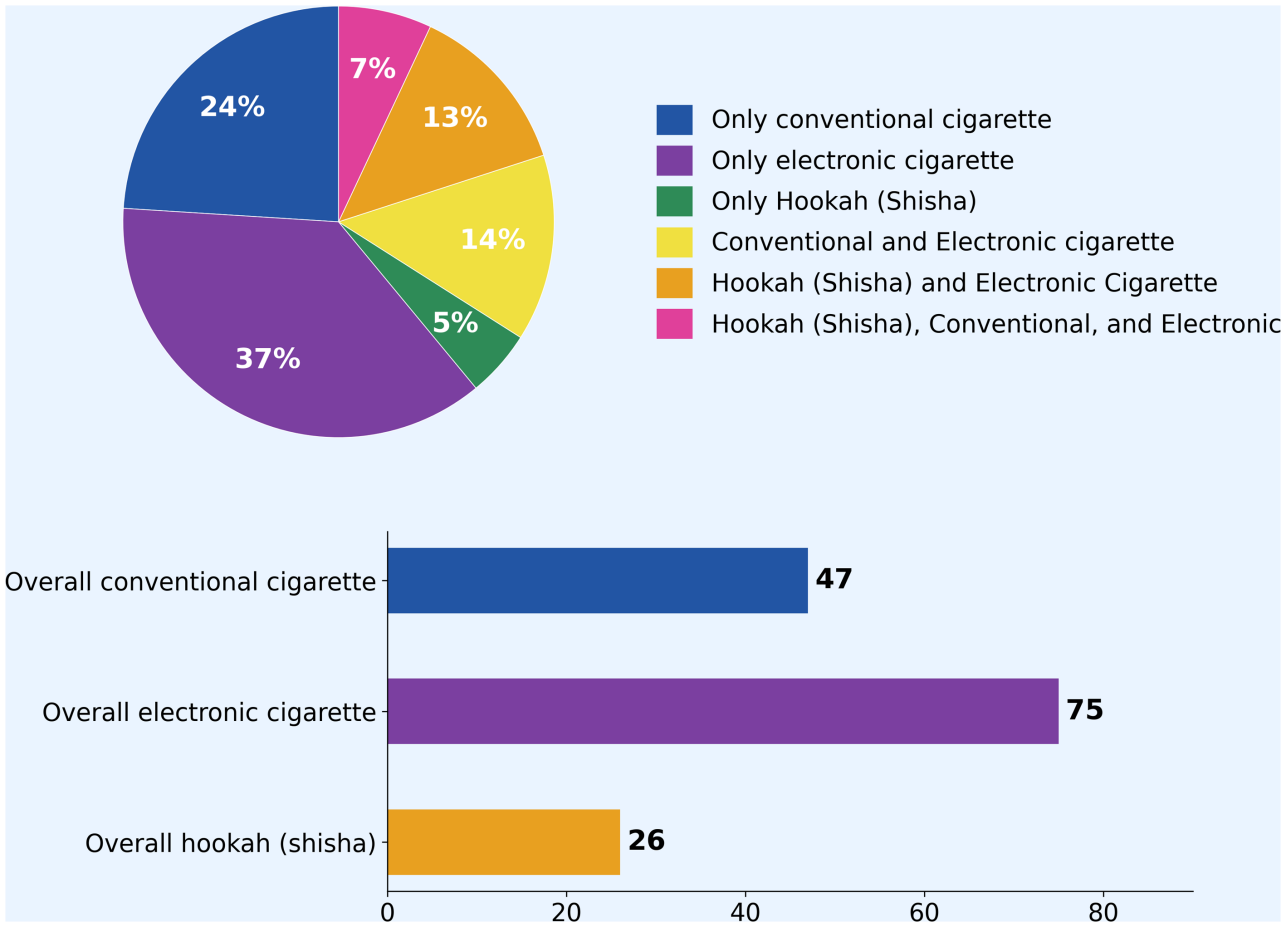
Types of smoking among current smokers (*n* = 96).

Among participants, 125 people (52.52%) never smoked, 15 (6.30%) reported former smoking status, 2 (0.84%) were occasional (social) smokers, and 96 (40.34%) were current smokers. Figure 1 demonstrates various types of smoking, using conventional cigarettes, Electronic E-cigarettes, and Hookah (Shisha). Smokers indicated different traditional and electronic cigarette brands (ranging from 1 to 17 distinct brands). However, 63 smokers use only one brand, 13 people smoke two brands of cigarettes, five individuals declare to smoke three brands, and single persons indicated more than three brands. Difficulty in refraining from smoking in places where it is forbidden (e.g., in a mosque, at the library, in a cinema, etc.) was reported by 38 people. In the sample, 37 participants reported smoking more frequently during the first hours after waking than during the rest of the day. Also, 51 respondents declared they feel stressed, nervous, restless, or anxious if they cannot smoke.

Associations between current smoking and other demographic and health-related variables were analyzed using contingency tables and Pearson’s χ^2^ test of independence (Table 3). More non-smokers were found among older participants than younger (*p* < 0.05). Significantly more women smoke compared to men (*p* < 0.001). Also, More smokers were present in people suffering from mental disorders than in mentally healthy respondents (*p* < 0.01).

**Table 3:** Associations of current smoking status with other variables.

Variable	Category	Non-smoker		Smoker		Χ^2^(1)	*p*	φ
		*n*	*%*	*n*	*%*			
Age	Younger (under 30)	82	34.45	71	29.83	4.84	0.028	−0.14
	Older (above 30)	58	24.37	27	11.35			
Sex	Male	91	38.24	25	10.50	35.98	< 0.001	0.39
	Female	49	20.59	73	30.67			
Relationship status	Married	13	5.46	3	1.26	3.56	0.059	0.12
	Single	127	53.36	95	39.92			
Job	Employed	66	27.73	47	19.75	0.02	0.901	−0.01
	Unemployed	74	31.09	51	21.43			
Disability	No	103	43.83	72	30.64	0.01	0.943	0.01
	Yes	35	14.89	25	10.64			
Asthma	No	127	53.36	86	36.13	0.54	0.464	0.05
	Yes	13	5.46	12	5.04			
Diabetes	No	133	55.88	93	39.08	0.00	0.972	0.00
	Yes	7	2.94	5	2.10			
Heart disease	No	134	56.30	92	38.66	0.41	0.524	0.04
	Yes	6	2.52	6	2.52			
Mental disorder	No	138	57.98	88	36.98	9.27	0.002	0.20
	Yes	2	0.84	10	4.20			
Neurological disorder	No	139	58.40	96	40.34	0.82	0.367	0.06
	Yes	1	0.42	2	0.84			
Immunological disease	No	134	56.30	97	40.76	2.15	0.142	−0.10
	Yes	6	2.52	1	0.42			
Other disease	No	133	55.88	90	37.82	0.98	0.323	0.06
	Yes	7	2.94	8	3.36			
Vaccine	No	136	57.14	94	39.50	0.27	0.606	0.03
	Yes	4	1.68	4	1.68			
Post-COVID	No	86	36.13	65	27.31	0.60	0.44	−0.05
	Yes	54	22.69	33	13.87			

### 3.4 Associations between COVID-19 and smoking

Associations between smoking behavior and the number of symptoms reported by participants during the COVID-19 infection were examined using an ordinal regression model. The initial diagnostic assessment supports the model’s adequacy. The overall model test was statistically significant, χ^2^(17) = 50.56, *p* < 0.001, indicating that the included predictors significantly improved the model fit over the intercept-only null model. Goodness-of-fit was assessed through pseudo *R*^2^ measures, with Nagelkerke *R*^2^_N_ = 0.054, reflecting a modest but consistent improvement in explanatory power as health and behavioral variables were added. The stability of the standard errors and the consistency of odds ratio directions across the three iterative models suggest no immediate evidence of model instability. The variance inflation factor (VIF) ranged from 1.15 to 3.46, which is an acceptable value and indicates that multicollinearity is not a problem. Gender and relationship status are related to a high number of COVID-19 symptoms. Men are two times more likely (*b* = 0.73, AOR = 2.07, *p* < 0.01) to suffer from many coronavirus symptoms than women, and singles (and widowed persons) are four times more susceptible to a high number of COVID-19 symptoms than married people (*b* = 1.40, AOR = 4.03, *p* < 0.01). Those with immunological diseases are less likely to experience many coronavirus symptoms (*b* = −1.76, AOR = 0.17, *p* < 0.05), but the association is weak. In addition, people who smoke more frequently in the morning than during the rest of the day are three and a half times more exposed to a high number of coronavirus symptoms, as shown in Table 4.

**Table 4:** Logistic regression analysis for symptoms of COVID-19.

Predictor variable	Symptoms of the COVID-19								
	Symptoms number			Symptoms severity			Post-Coronavirus		
	*b*	*SE*	AOR	*b*	*SE*	AOR	*b*	*SE*	AOR
Age above 30 (ref. under 30)	0.11	0.35	1.12	0.26	0.36	1.30	0.16	0.45	1.18
Male sex (ref. female)	0.73**	0.27	2.07	0.41	0.28	1.50	0.84*	0.35	2.32
Single status (ref. married)	1.40**	0.52	4.03	0.42	0.56	1.52	1.10	0.73	3.00
Unemployed (ref. employed)	0.53	0.33	1.70	0.07	0.33	1.07	−0.14	0.42	0.87
Disability	−0.22	0.29	0.81	−0.43	0.31	0.65	−1.56***	0.43	0.21
Asthma	0.11	0.40	1.11	0.04	0.43	1.04	1.95*	0.90	7.05
Diabetes	−0.78	0.64	0.46	−0.69	0.62	0.50	1.65	0.96	5.18
Heart disease	1.04	0.65	2.83	0.94	0.64	2.57	−0.90	0.95	0.41
Mental disorder	0.85	0.57	2.33	0.52	0.59	1.68	1.19	0.90	3.29
Neurological disorder	−1.45	1.10	0.23	−1.52	1.24	0.22	−13.75	14.67	0.00
Immunological disease	−1.76*	0.79	0.17	−2.14**	0.83	0.12	−17.09	16.46	0.00
Current smoking status	−0.61	0.43	0.54	−0.93*	0.44	0.40	−0.32	0.56	0.73
Types of smoking	0.18	0.26	1.20	0.31	0.27	1.36	−0.19	0.35	0.83
Cigarette brands	−0.11	0.10	0.89	−0.14	0.12	0.87	0.08	0.17	1.08
Difficulty to not smoking	0.16	0.42	1.17	0.20	0.43	1.22	−0.53	0.55	0.59
Morning smoking	1.28**	0.45	3.58	1.03*	0.45	2.81	0.98	0.55	2.66
Nervousness if cannot smoke	0.07	0.42	1.08	0.42	0.42	1.51	0.25	0.52	1.29

*p < 0.05, **p < 0.01, ***p < 0.001.

The model diagnostics support the validity of the ordinal regression model, conducted to test associations between smoking behavior and self-reported severity of COVID-19 symptoms. The overall model test was statistically significant, χ^2^(17) = 34.44, *p* = 0.007, demonstrating that the full set of predictors significantly improved model fit compared to the null model. Goodness-of-fit improved across the iterations, with the Nagelkerke *R*^2^_N_ reaching 0.054. The standard errors for the predictors remained stable across all three models, and VIF < 5, suggesting that multicollinearity did not adversely affect the coefficient estimates. Additionally, the consistent direction and magnitude of the odds ratios across model specifications provide further evidence of the model’s reliability. Symptoms’ severity was negatively predicted by immunological disease (*b* = −2.14, AOR = 0.12, *p* < 0.05), but, as in previous results, the association was very weak (Table 4). Among smoking-related variables, the positive predictor of symptom severity was morning smoking need, while the negative predictor was current smoking status. People who smoke more frequently in the morning than in the evening are almost three times more likely to suffer severe symptoms of COVID-19 (*b* = 1.03, AOR = 2.81, *p* < 0.05). In contrast, those who currently smoke are almost half as likely to experience severe coronavirus symptoms (*b* = −0.93, AOR = 0.40, *p* < 0.05).

The binary logistic regression model was performed to assess relationships between smoking and reporting post-coronavirus symptoms. The initial diagnostics confirmed the statistical adequacy and fit of the models. The regression model significantly improved upon the null model χ^2^ (19) = 45.09, *p* < 0.001, and achieved a Nagelkerke *R*^2^_N_ of 0.24, indicating a substantial increase in explained variance compared to the baseline. Collinearity diagnostics demonstrated that all predictors maintained VIF well below the threshold of 5. Overall, the stability of the remaining VIF values and the significant omnibus tests support the model’s structural validity for the regression model. Post-coronavirus symptoms are two times more likely among men (*b* = 0.84, AOR = 2.32, *p* < 0.05) than women (Table 4) and seven times more likely in people who have asthma (*b* = 1.95, AOR = 7.05, *p* < 0.05). In contrast, disability seems to be a protective factor post-COVID-19 (*b* = −1.56, AOR = 0.21, *p* < 0.001).

## 4 Discussion

In this work, all participants had a history of coronavirus infection; a few were vaccinated. Regarding the characteristics of COVID-19 symptoms across all four infection periods, we found that most cases in the fourth infection period were asymptomatic and had a shorter disease duration. Ordinal regression studies indicated that men were two times more likely to exhibit coronavirus symptoms than women, and singles were four times more susceptible compared to married individuals. A weak negative correlation was observed between the presence of immunological diseases and the severity of coronavirus symptoms. Our Pearson’s analysis showed that non-smokers prevailed in older individuals compared to younger participants, while more women and individuals suffering from mental disorders smoke. The binary logistic regression model indicated that post-coronavirus symptoms were more prevalent among men and people with asthma. On the other hand, having a disability is a protective factor post-COVID-19. Yet, our findings may reflect residual confounding, selection bias, reporting bias, or other limitations of the study design, rather than a functional protective association of smoking and COVID-19 severity.

We found that individuals who frequently smoke in the morning compared to the rest of the day are at higher risk of expressing coronavirus symptoms. A previous report has found a link between nicotine dependence and the inability to retain abstinence with the time to consume the first cigarette in the morning (27). Besides, a systematic review reported that earlier time to the first cigarette is a significant indicator of tobacco dependence. An earlier time to the first cigarette was found to be an indication of higher chances for failure of smoking cessation and relapse (28).

In support of our findings, a meta-analysis reported that male gender is a risk factor for COVID-19 severity (29). A review has shown that biologically, the male gender was linked to an almost twofold elevated mortality risk, which gender-based immunopathological factors could drive (30). A meta-analysis examined factors related to worsening COVID-19 outcomes and found that men were at higher risk of both mortality and COVID-19 severity (31). In a multicenter study, progression of post-COVID-19 symptoms was worse in males than in females. Also, the plasma level of neutralizing antibodies was higher in men than in women (32). The gender-based differences could be due to biological differences in the immune system (33), but they may also be driven by social behavior (34, 35).

We found that single individuals were more susceptible to post-coronavirus symptoms than married individuals. Differences in marital status have been examined in the context of the COVID pandemic from different angles, including the psychological aspects. For instance, a previous study investigated psychological elements of the COVID-19 lockdown. They found that life satisfaction was higher in married couples than in single individuals (36, 37). A previous report has shown that the well-being and resilience of married individuals are protected during the first wave. Highlighting the role of marital status during the COVID-19 pandemic (38).

A previous report analyzed the dynamics of the COVID-19 infection and documented that mood disorders, including anxiety and depression, have increased (39). This is consistent with previous studies by researchers (40–42), including our team (43–45), and highlights the paramount need to establish awareness and policies targeting mental health. Furthermore, mental well-being should be integrated into response strategies for COVID-19 cases, including monitoring psychological needs and providing support (40). Besides, our findings indicated that the rate of smoking was higher among participants with mental health issues. This observation aligns with existing evidence demonstrating that the majority of psychiatric patients are smokers (46–48).

We reported a higher percentage of symptom-free individuals and shorter disease duration with subsequent infections. Consistent with our results, subsequent infections exhibit a milder pattern than the first. A recent UK report, which included more than 40,000 reinfections, found that viral load was lower in reinfections. Moreover, the percentages of self-reported symptoms were notably lower (49). This could also be attributed to another factor: different COVID-19 waves. The characteristics of COVID-19’s four waves indicate variation in the reported severity, duration, and hospitalization time. These dynamics have been reported previously (50–52). Tracking COVID-19 variants, including alpha, beta, gamma, and delta, has shown that none are clinically concerning. These variants have been de-escalated from significant threat status (53). Compared with the first wave, the number of symptomatic pediatric patients in the second and third waves has decreased (52). In line with this, a retrospective study examined the clinical presentation and outcomes in hospitalized Saudi individuals. The study showed that the mortality rate was lower in the second wave than in the first (54).

Our findings indicated that current smokers prevailed in electronic cigarette consumers compared to other types of smoking. This could be rationalized by the notion that electronic cigarettes were initially promoted as safe and smoking cessation aids (55, 56). Regarding the gender-based analysis of current smoking, we found that females outnumber males in this comparison. In support of this, a previous study has shown that gender differences are significant in the consumption of electronic cigarettes. About 40% of females were more likely to use electronic cigarettes, whereas the probability of men trying it reached 30% (57). This could be driven by the fact that men smoke primarily to maintain positive reinforcement emotions such as reward, pleasure, and enjoyment. At the same time, women smoke to distract from negative reinforcement emotions such as anxiety and stress (58).

Another implication is that this evidence encourages the promotion of innovative solutions to prevent smoking, especially in women and the younger population. Further, provide a smoking cessation program. These initiatives would promote health and reduce the economic burden, especially during respiratory pandemics such as COVID-19 and SARS (59).

Current smoking status negatively predicts Symptom severity. This could be attributed to the fact that non-smoker individuals were older than current smokers. Besides, we found that the majority of current smokers consume electronic cigarettes, which is more common in the younger population (60). Age is well-recognized as an independent risk factor for COVID-19 mortality (23). As people age, they exhibit health conditions that may deteriorate with smoking. Thus, most aged individuals try to quit and become former smokers (61). Our study design has multiple limitations that could contribute to the observed negative association, such as selection bias, response fatigue bias, reporting bias, and a small sample size. These methodological characteristics may influence the observed association rather than indicating a fundamental negative association.

Further, we found that individuals suffering from mental disorders smoke more compared to mentally healthy individuals. We did not have an identifiable profile of mental disorders that these smokers exhibit. Yet, accumulated evidence supports that the prevalence of nicotine smoking is relatively high in individuals with mental disorders (62–65). It was reported that individuals who suffer from mental disorders have a twofold chance of smoking compared to individuals with no mental disorders (63).

Our regression analyses indicated that asthmatics had higher post-coronavirus symptom scores. A previous report conducted in a multicenter Japanese population supported these findings. The study revealed that asthma is a risk factor for prolonged post-coronavirus symptoms. In a 1-year follow-up, univariate logistic regression analyses indicated an association between protracted post-coronavirus fatigue and asthma (66). A systematic review has shown that allergic diseases such as asthma and rhinitis are risk factors for prolonged coronavirus symptoms. Based on reviewing 13 prospective cohort studies, pre-existing asthma and rhinitis are associated with significantly elevated risks of long-term COVID-19 symptoms (67).

We observed that disability served as a protective factor against post-COVID-19 outcomes. Yet, our observation is limited because our sample size is relatively small and we do not have specific identification of respondents’ disability conditions. The types and prevalence of disabilities vary, including visual, hearing, mobility, and cognitive impairments (68). On the other hand, previous reports indicated that individuals with disabilities presented resilience compared to people without disabilities during Hurricane Katrina; this level of protection could be attributed to the existence of a support system for individuals with disabilities before the emergence of an issue (69).

Our findings support previous studies linking smoking to COVID-19 severity, including meta-analyses and systematic reviews (16, 70), as well as Mendelian and observational study designs (71). Berlin et al. (72) examined the link between smoking and COVID-19. They reported the need for further studies in this domain to support and strengthen these findings, with the aim of improving health and well-being during the pandemic. Collectively, with our findings, it is essential to translate and incorporate this knowledge to (1) facilitate the establishment of evidence-based health-sustainable policies and (2) achieve SDGs (73). For example, Alqahtani et al., in their report, summarized strategic interventions that could be beneficial in smoking cessation practice, such as (1) extending benefits for the healthcare professional who provides smoking cessation services, (2) facilitating approaching nicotine replacement products remotely, (3) identifying smoking cessation services as a quality measure for healthcare systems accreditation, which would augment efforts toward smoking cessation, (4) Utilize channels that are fundamental in influencing smoking decisions and behavior (59).

Limitations of the study: Our study is cross-sectional, which limits the ability to infer causal relationships between the study variables. A longitudinal cohort with a bigger sample size would prevent this limitation. Another limitation of our study design is the recall bias. Further, our sample size is adequate, and the association was significant, but the factor was weak. Thus, a larger sample would be advantageous. Additionally, selection bias may have occurred due to convenience sampling and the distribution of the online survey, potentially limiting the sample’s representativeness. Besides, the study sample is heavily skewed toward young, male student populations. This could be attributed to the sampling design that used social media, in which selection was based on digital algorithms and interests. Also, our findings may not reflect the true prevalence of COVID-19.

Moreover, we did not collect detailed information about the type of disability. Future prospective studies that build on in-person interviews could help address these limitations. Another factor is the misunderstanding of vaccination-related questions, particularly those about multiple doses, which can be confusing. Despite adjusting for multiple demographic and health-related variables, the possibility of residual confounding remains. Unmeasured factors such as socioeconomic status, healthcare access, or unreported comorbidities could influence the observed associations between smoking and COVID-19 outcomes.

Collider bias is also a concern, particularly if smoking status and COVID-19 severity both influence participation likelihood or reporting, which could distort the observed relationships. Behavioral factors might also contribute to the findings. For example, underreporting of symptoms could differ by smoking status, gender, or relationship status, potentially due to social desirability bias or differential health awareness. Similarly, disparities in healthcare access or health-seeking behaviors may affect symptom reporting and severity assessment, especially in subpopulations such as singles or individuals with mental disorders. These factors could influence both exposure and outcome measures and should be considered when interpreting the results. Response fatigue bias is an additional factor that may affect the accuracy and quality of the findings.

Future studies employing longitudinal designs with randomized sampling and objective clinical assessments are needed to minimize these biases. Incorporating detailed socioeconomic and healthcare access data would allow better control of confounding. Additionally, triangulating self-reported data with medical records could reduce underreporting and improve accuracy.

Another limitation of our study is the use of composite scores that sum symptom counts and severity across multiple COVID-19 infection episodes. While this approach provides an overall estimate of symptom burden, it may obscure important temporal dynamics and variations in symptomatology across different infection waves. Aggregating symptoms across infections treats all episodes as a single event, potentially introducing measurement ambiguity and limiting the ability to detect changes in symptom severity or profiles over time. Future research employing longitudinal or repeated-measures designs is warranted to more precisely examine the temporal evolution of symptoms and severity following reinfections.

## 5 Conclusion

Our findings indicated that among smoking types, the electronic cigarette is emerging, especially in young women. Also, being single and male, they are more susceptible to coronavirus symptoms. Further, consistent with previous studies, we found that post-coronavirus symptoms were higher in men and in individuals with asthma. Therefore, it is paramount to establish a continuous preparedness and recovery policy that addresses this population’s vulnerabilities and risks. The need to establish a sustainable emergency response system based on the COVID-19 experience is unmet at the national level.

## Data Availability

The original data are available from the corresponding author upon reasonable request.

## Footnotes

1
https://www.psychiatry.org/psychiatrists/practice/dsm
