# Supplementary material for: The association between smoking and COVID-19 symptoms, severity, and post-COVID-19 symptoms: a cross-sectional survey study
Source: Front Public Health. 2026 Jul 6;14:1746370. doi: 10.3389/fpubh.2026.1746370 (PMC13381621; doi:10.3389/fpubh.2026.1746370)
Supplement: Supplementary file 1 [file Table_1.docx]

The association between smoking and COVID-19 symptoms, severity, and post-COVID-19 symptoms, a Cross-Sectional survey study- supplementary material.

Survey:

The survey included multiple questions representing different aspects and will be distributed via [REDCap](https://ksusa-my.sharepoint.com/personal/malshammari_ksu_edu_sa/Documents/KSU%20Saud%20Home%20and%20Office%202B100/Projects/E-Cig-&%20Traditional%20Smoking/rcmed.ksu.edu.sa/) (Vanderbilt University, USA). Survey items include.

1. Demographic information
2. History and type of conventional smoking and E-Cig
3. Smoking frequency (Smoking Dependency)
4. Comorbidity
5. COVID-19 Severity.

Participants provided information on sex, age, state of residence, residence, employment, and education. The estimated completion time of the survey will be 10-15 minutes.

**Appendix 1**

| **The Questionnaire** | |  |
| --- | --- | --- |
| **Variable(s)** | **values** |  |
| Age | \| 1 \| < 18 \| \| --- \| --- \| \| 2 \| 18-30 \| \| 3 \| 30-45 \| \| 4 \| > 45 \| | |
| Sex | \| 1 \| Male \| \| --- \| --- \| \| 2 \| Female \| | |
| Marital Status | \| 1 \| Single \| \| --- \| --- \| \| 2 \| Married \| \| 3 \| Widow/widower \| \| 4 \| Divorced \| | |
| Current Status | \| 1 \| Student \| \| --- \| --- \| \| 2 \| Employee \| \| 3 \| Housewife \| \| 4 \| Other \| | |
| Please specify | text | |
| Occupation Status | \| 1 \| Governmental employment \| \| --- \| --- \| \| 2 \| Private sector \| \| 4 \| Unemployed \| \| 5 \| Long-term unemployed \| \| 6 \| Under retirement \| \| 7 \| Others \| | |
| Nationality | \| 1 \| Saudi \| \| --- \| --- \| \| 2 \| None Saudi \| | |
| Do you have a disability ? | \| 1 \| Yes \| \| --- \| --- \| \| 0 \| No \| | |
| Do you have a long-standing illness? | \| 1 \| Asthma \| \| --- \| --- \| \| 2 \| Diabetes \| \| 3 \| Hypertension or heart disease \| \| 4 \| Mental disorders \| \| 5 \| Neurological disorders \| \| 6 \| Immunological disease (arthritis, Systemic lupus erythematosus ..ect..) \| \| 7 \| Others \| \| 8 \| No long -standing illness \| | |
| How many year(s) do you suffer from Asthma since the diagnosis? | \| 1 \| Less than 1 year \| \| --- \| --- \| \| 2 \| 2 years \| \| 3 \| 3 years \| \| 4 \| 5 years \| \| 5 \| More than 5 years \| | |
| How many year(s) do you suffer from Diabetes since the diagnosis? | \| 1 \| Less than 1 year \| \| --- \| --- \| \| 2 \| 2 years \| \| 3 \| 3 years \| \| 4 \| 5 years \| \| 5 \| More than 5 years \| | |
| How many year(s) do you suffer from Hypertension or Heart Diseases since the diagnosis? | \| 1 \| Less than 1 year \| \| --- \| --- \| \| 2 \| 2 years \| \| 3 \| 3 years \| \| 4 \| 5 years \| \| 5 \| More than 5 years \| | |
| How many year(s) do you suffer from Mental Disorders since the diagnosis? | \| 1 \| Less than 1 year \| \| --- \| --- \| \| 2 \| 2 years \| \| 3 \| 3 years \| \| 4 \| 5 years \| \| 5 \| More than 5 years \| | |
| How many year(s) do you suffer from Neurological Disorders since the diagnosis? | \| 1 \| Less than 1 year \| \| --- \| --- \| \| 2 \| 2 years \| \| 3 \| 3 years \| \| 4 \| 5 years \| \| 5 \| More than 5 years \| | |
| How many year(s) do you suffer from Immunological Diseases since the diagnosis? | \| 1 \| Less than 1 year \| \| --- \| --- \| \| 2 \| 2 years \| \| 3 \| 3 years \| \| 4 \| 5 years \| \| 5 \| More than 5 years \| | |
| Please write it/them here | text | |
| Geographical region | \| 1 \| The Central Region \| \| --- \| --- \| \| 2 \| The Western Region \| \| 3 \| The Eastern Region \| \| 4 \| The Southern Region \| \| 5 \| The Northern Region \| | |
| Section Header: *COVID-19 status_1*  COVID-19 status | \| 1 \| Confirmed positive \| \| --- \| --- \| \| 2 \| Not at all \| | |
| Have you been vaccinated against COVID-19? | \| 1 \| Yes \| \| --- \| --- \| \| 0 \| No \| | |
| How many doses have you taken? | \| 1 \| One dose \| \| --- \| --- \| \| 2 \| Two doses \| \| 3 \| Three doses \| | |
| First vaccine date | text (date_dmy) | |
| Second vaccine date | text (date_dmy) | |
| Third vaccine date | text (date_dmy) | |
| First vaccine type | \| 1 \| (Pfizer-BioNTech) \| \| --- \| --- \| \| 2 \| (Moderna) \| \| 3 \| (Oxford-Astrazeneca) \| \| 4 \| (Janssen) \| \| 5 \| Other \| | |
| Second vaccine type | dropdown   \| 1 \| (Pfizer-BioNTech) \| \| --- \| --- \| \| 2 \| (Moderna) \| \| 3 \| (Oxford-Astrazeneca) \| \| 4 \| (Janssen) \| \| 5 \| Other \| | |
| Third vaccine type | \| 1 \| (Pfizer-BioNTech) \| \| --- \| --- \| \| 2 \| (Moderna) \| \| 3 \| (Oxford-Astrazeneca) \| \| 4 \| (Janssen) \| \| 5 \| Other \| | |
| If you were confirmed positive | \| 1 \| Once \| \| --- \| --- \| \| 2 \| Twice \| \| 3 \| Three times \| \| 4 \| Four times \| | |
| Date of your first infection | text (date_dmy) | |
| Date of your second infection | text (date_dmy) | |
| Date of your third infection | text (date_dmy) | |
| Date of your fourth infection | text (date_dmy) | |
| Hospitalization status for first COVID-19 infection | \| 1 \| Two days or less \| \| --- \| --- \| \| 2 \| 2-5 days \| \| 3 \| 5-9 days \| \| 4 \| More than 9 days \| \| 5 \| Not hospitalized \| | |
| Hospitalization status for second COVID-19 infection? | \| 1 \| Two days or less \| \| --- \| --- \| \| 2 \| 2-5 days \| \| 3 \| 5-9 days \| \| 4 \| More than 9 days \| \| 5 \| Not hospitalized \| | |
| Hospitalization status for third COVID-19 infection | \| 1 \| Two days or less \| \| --- \| --- \| \| 2 \| 2-5 days \| \| 3 \| 5-9 days \| \| 4 \| More than 9 days \| \| 5 \| Not hospitalized \| | |
| Hospitalization status for fourth COVID-19 infection | \| 1 \| Two days or less \| \| --- \| --- \| \| 2 \| 2-5 days \| \| 3 \| 5-9 days \| \| 4 \| More than 9 days \| \| 5 \| Not hospitalized \| | |
| Hospitalization visit Status for first infection | \| 1 \| Intensive Care Unit (ICU) \| \| --- \| --- \| \| 2 \| Regular hospital ward \| \| 3 \| Emergency visit and observation \| \| 4 \| Other \| | |
| Hospitalization visit status for second infection | \| 1 \| Intensive Care Unit (ICU) \| \| --- \| --- \| \| 2 \| Regular hospital ward \| \| 3 \| Emergency visit and observation \| \| 4 \| Other \| | |
| Hospitalization visit status for third infection | \| 1 \| Intensive Care Unit (ICU) \| \| --- \| --- \| \| 2 \| Regular hospital ward \| \| 3 \| Emergency visit and observation \| \| 4 \| Other \| | |
| Hospitalization visit status for fourth infection | \| 1 \| Intensive Care Unit (ICU) \| \| --- \| --- \| \| 2 \| Regular hospital ward \| \| 3 \| Emergency visit and observation \| \| 4 \| Other \| | |
| What symptoms did you have during first infection: (You can choose more than one option) | \| 1 \| Fever \| \| --- \| --- \| \| 2 \| Cough \| \| 3 \| Tiredness \| \| 4 \| Loss of taste or smell \| \| 5 \| Shortness of breath or difficulty breathing \| \| 6 \| Muscle aches \| \| 7 \| Other \| \| 8 \| No Symptoms \| | |
| What symptoms did you have during second infection: (You can choose more than one option) | \| 1 \| Fever \| \| --- \| --- \| \| 2 \| Cough \| \| 3 \| Tiredness \| \| 4 \| Loss of taste or smell \| \| 5 \| Shortness of breath or difficulty breathing \| \| 6 \| Muscle aches \| \| 7 \| Other \| \| 8 \| No Symptoms \| | |
| What symptoms did you have during third infection: (You can choose more than one option) | \| 1 \| Fever \| \| --- \| --- \| \| 2 \| Cough \| \| 3 \| Tiredness \| \| 4 \| Loss of taste or smell \| \| 5 \| Shortness of breath or difficulty breathing \| \| 6 \| Muscle aches \| \| 7 \| Other \| \| 8 \| No Symptoms \| | |
| What symptoms did you have during fourth infection: (You can choose more than one option) | \| 1 \| Fever \| \| --- \| --- \| \| 2 \| Cough \| \| 3 \| Tiredness \| \| 4 \| Loss of taste or smell \| \| 5 \| Shortness of breath or difficulty breathing \| \| 6 \| Muscle aches \| \| 7 \| Other \| \| 8 \| No Symptoms \| | |
| How severe are these symptoms for the first infection? | \| 1 \| Mild \| \| --- \| --- \| \| 2 \| Moderate \| \| 3 \| Severe \| | |
| How severe are these symptoms for the second infection? | \| 1 \| Mild \| \| --- \| --- \| \| 2 \| Moderate \| \| 3 \| Severe \| | |
| How severe are these symptoms for the third infection? | radio   \| 1 \| Mild \| \| --- \| --- \| \| 2 \| Moderate \| \| 3 \| Severe \| | |
| How severe are these symptoms for the fourth infection ? | \| 1 \| Mild \| \| --- \| --- \| \| 2 \| Moderate \| \| 3 \| Severe \| | |
| How long have these symptoms lasted for the first infection? | \| 1 \| Five days or less \| \| --- \| --- \| \| 2 \| 6 days to 2 weeks \| \| 3 \| More than two weeks to a month \| \| 4 \| More than a month \| | |
| How long have these symptoms lasted for the second infection? | radio   \| 1 \| Five days or less \| \| --- \| --- \| \| 2 \| 6 days to 2 weeks \| \| 3 \| More than two weeks to a month \| \| 4 \| More than a month \| | |
| How long have these symptoms lasted for the third infection? | radio   \| 1 \| Five days or less \| \| --- \| --- \| \| 2 \| 6 days to 2 weeks \| \| 3 \| More than two weeks to a month \| \| 4 \| More than a month \| | |
| How long have these symptoms lasted for the fourth infection? | radio   \| 1 \| Five days or less \| \| --- \| --- \| \| 2 \| 6 days to 2 weeks \| \| 3 \| More than two weeks to a month \| \| 4 \| More than a month \| | |
| Do you suffer from Post-COVID Conditions? | \| 1 \| Yes \| \| --- \| --- \| \| 0 \| No \| | |
| Please write it/them here | text | |
| Section Header: *Smoking Questions*  Are you a smoker/ former smoker? | \| 1 \| Yes \| \| --- \| --- \| \| 0 \| No \| | |
| Smoking Status | radio   \| 1 \| Current Smoker \| \| --- \| --- \| \| 2 \| Former \| \| 3 \| Occasional smoker / social smoker \| | |
| After acquiring COVID-19 have your smoking pattern or frequency changed? | \| 1 \| Yes \| \| --- \| --- \| \| 0 \| No \| | |
| What kind of cigarettes do/did you usually use? | radio, Required   \| 1 \| Conventional cigarette \| \| --- \| --- \| \| 2 \| Electronic cigarette \| \| 3 \| Conventional cigarette and Electronic \| \| 4 \| Hookah (Shisha) \| \| 5 \| Hookah (Shisha) & Electronic Cigarette \| \| 6 \| All above \| | |
| Conventional Cigarette type? | checkbox   \| 1 \| Marlboro \| \| --- \| --- \| \| 2 \| Davidoff \| \| 3 \| Dunhill \| \| 4 \| Kent \| \| 5 \| L&M \| \| 6 \| Rothman \| \| 7 \| Benson & Hedges \| \| 8 \| Chesterfield \| \| 9 \| Monte Carlo \| \| 10 \| Pall Mall \| \| 11 \| Parliament \| \| 12 \| Safeway \| \| 13 \| Salem \| \| 14 \| Vogue \| \| 15 \| Wenpor \| \| 16 \| West \| \| 17 \| Winston \| \| 18 \| I prefer to not mention \| \| 19 \| Other \| | |
| Other | text | |
| Nicotine Percentage? | radio   \| 1 \| 1-10 mg \| \| --- \| --- \| \| 2 \| 11-20 mg \| \| 3 \| 21-30 mg \| \| 4 \| 31-50 mg \| \| 5 \| >50 mg \| \| 6 \| Other \| \| 7 \| I don't know \| | |
| Please Specify | text | |
| How soon after you wake up do you smoke your first cigarette? | radio   \| 1 \| Within 5 minutes \| \| --- \| --- \| \| 2 \| 6-30 minutes \| \| 3 \| 31-60 minutes \| \| 4 \| After 60 minutes \| \| 5 \| other \| | |
| Other | text | |
| Do you find it difficult to refrain from smoking in places where it is forbidden, e.g., in church, at the library, in a cinema, etc.? | radio   \| 1 \| Yes \| \| --- \| --- \| \| 2 \| No \| | |
| Which cigarette would you hate most to give up? | radio   \| 1 \| The first one in the morning \| \| --- \| --- \| \| 2 \| The rest of the day \| | |
| Do you smoke more frequently during the first hours after waking than during the rest of the day? | radio   \| 1 \| Yes \| \| --- \| --- \| \| 2 \| No \| | |
| How many cigarettes per day do you smoke? | radio   \| 1 \| 10 or less \| \| --- \| --- \| \| 2 \| 11-20 \| \| 3 \| 21-30 \| \| 4 \| 31 or more \| | |
| Do you smoke if you are sick and bedridden? | radio   \| 1 \| Yes \| \| --- \| --- \| \| 2 \| No \| | |
| During the days when you can smoke freely, when do you smoke the first cigarette of the day? | radio   \| 1 \| 0 - 5 mins \| \| --- \| --- \| \| 2 \| 6 - 15 mins \| \| 3 \| 16 - 30 mins \| \| 4 \| 31 - 60 mins \| \| 5 \| More than 60 mins \| | |
| Do you sometimes awaken at night to have a cigarette? | radio   \| 1 \| Yes \| \| --- \| --- \| \| 2 \| No \| | |
| If yes how many nights in a week you wake up to smoke? | radio   \| 1 \| 0-1 nights \| \| --- \| --- \| \| 2 \| 2 or three nights \| \| 3 \| More than 4 nights \| | |
| Do you smoke now because it is really hard to quit? | radio   \| 1 \| Yes \| \| --- \| --- \| \| 2 \| No \| | |
| Do you ever have strong cravings to smoke? | radio   \| 1 \| Yes \| \| --- \| --- \| \| 2 \| No \| | |
| Over the past week, how strong have the urges to smoke been? | radio   \| 1 \| None/Slight \| \| --- \| --- \| \| 2 \| Moderate/Strong \| \| 3 \| Very Strong/Extremely Strong \| | |
| Is it hard to not to smoke in places where smoking is not allowed? | radio   \| 1 \| Yes \| \| --- \| --- \| \| 2 \| No \| | |
| When you haven't used conventional smoking for a while or when you're trying to stop using it, do you have tantrums because of your resistance to smoking? | radio   \| 1 \| Yes \| \| --- \| --- \| \| 2 \| No \| | |
| Do you feel stressed or anxious when not smoking conventional cigarette? | yesno   \| 1 \| Yes \| \| --- \| --- \| \| 0 \| No \| | |
| E-cigarette Type? | checkbox   \| 1 \| Air Bar \| \| --- \| --- \| \| 2 \| Ismod Nano \| \| 3 \| Nuso \| \| 4 \| Vuse \| \| 5 \| Relx \| \| 6 \| JUUL \| \| 7 \| MAZAJ \| \| 8 \| Puff Bar \| \| 9 \| MarkTen \| \| 10 \| Blu and Logic \| \| 11 \| SMOK \| \| 12 \| Prefer not to mention \| \| 13 \| Other \|   Custom alignment: LV | |
| Other | text | |
| Nicotine Percentage? | radio   \| 1 \| 1-10 mg/mL \| \| --- \| --- \| \| 2 \| 11-20 mg/mL \| \| 3 \| 21-30 mg/mL \| \| 4 \| 31-50 mg/mL \| \| 5 \| >50 mg/mL \| \| 6 \| Other \| \| 7 \| I don't know \| | |
| Please Specify | text | |
| How soon after you wake up do you smoke your first cigarette? | radio   \| 1 \| Within 5 minutes \| \| --- \| --- \| \| 2 \| 6-30 minutes \| \| 3 \| 31-60 minutes \| \| 4 \| After 60 minutes \| \| 5 \| other \| | |
| Please specify | text | |
| Do you find it difficult to refrain from smoking [E-cigarette] in places where it is forbidden, e.g., at the library, in a cinema, etc.? | radio   \| 1 \| Yes \| \| --- \| --- \| \| 2 \| No \| | |
| Which cigarette would you hate most to give up [electronic cigarette smoking]? | radio   \| 1 \| The first one in the morning \| \| --- \| --- \| \| 2 \| All others \| | |
| Do you smoke [ electronic cigarette]more frequently during the first hours after waking than during the rest of the day? | yesno   \| 1 \| Yes \| \| --- \| --- \| \| 0 \| No \| | |
| How many cigarettes per day do you usually smoke [use your electronic cigarette]? ([assume that one "time" consists of around 15 puffs or lasts around 10 minutes]) | radio   \| 1 \| 150 puffs or less \| \| --- \| --- \| \| 2 \| 160-200 puffs \| \| 3 \| 201- 300 puffs \| \| 4 \| More than 300 puffs \| \| 5 \| Other than above \| | |
| Please specify | text | |
| How many cigarettes per day do you usually smoke? ([assume that one "time" consists of around 15 puffs or lasts around 10 minutes]) | radio   \| 1 \| 0-4 times/day \| \| --- \| --- \| \| 2 \| 5-9 times/day \| \| 3 \| 10-14 times/day \| \| 4 \| 15-19 times/day \| \| 5 \| 20-29 times/day \| \| 6 \| More than 30 times/day \| | |
| Do you smoke [electronic cigarette] if you are sick and bedridden? | radio   \| 1 \| Yes \| \| --- \| --- \| \| 2 \| No \| | |
| On days that you can smoke [use your electronic cigarette] freely, how soon after you wake up do you smoke your first cigarette of the day [first use your electronic cigarette]? | radio   \| 1 \| Within 5 minutes \| \| --- \| --- \| \| 2 \| 6-30 minutes \| \| 3 \| 31-60 minutes \| \| 4 \| After 60 minutes \| | |
| Do you sometimes awaken at night to have a cigarette [use your electronic cigarette]? | radio   \| 1 \| Yes \| \| --- \| --- \| \| 2 \| No \| | |
| If yes, how many nights per week do you typically awaken to smoke [use your electronic cigarette]? | radio   \| 1 \| 0-1 nights \| \| --- \| --- \| \| 2 \| 2-3 nights \| \| 3 \| 4+ nights \| | |
| Do you smoke [use an electronic cigarette] now because it is really hard to quit? | radio   \| 1 \| Yes \| \| --- \| --- \| \| 2 \| No \| | |
| Do you ever have strong cravings to smoke [use an electronic cigarette]? | radio   \| 1 \| Yes \| \| --- \| --- \| \| 2 \| No \| | |
| Is it hard to keep from smoking [using an electronic cigarette] in places where you are not supposed to? | radio   \| 1 \| Yes \| \| --- \| --- \| \| 2 \| No \| | |
| Over the past week, how strong have the urges to smoke [use an electronic cigarette] been? | radio   \| 1 \| None/Slight \| \| --- \| --- \| \| 2 \| Moderate/Strong \| \| 3 \| Very Strong/Extremely Strong \| | |
| When you haven't used tobacco [an electronic cigarette] for a while or when you tried to stop smoking [using]... Do you feel more irritable because you couldn't smoke [use an electronic cigarette]? | yesno   \| 1 \| Yes \| \| --- \| --- \| \| 0 \| No \| | |
| Do you feel nervous, restless, or anxious because you couldn't smoke [use an electronic cigarette]? | radio   \| 1 \| Yes \| \| --- \| --- \| \| 2 \| No \| | |
| What type of conventional/electronic cigarette? | checkbox   \| 1 \| Marlboro \| \| --- \| --- \| \| 2 \| Davidoff \| \| 3 \| Dunhill \| \| 4 \| Kent \| \| 5 \| L&M \| \| 6 \| Rothman \| \| 7 \| Benson & Hedges \| \| 8 \| Chesterfield \| \| 9 \| Monte Carlo \| \| 10 \| Pall Mall \| \| 11 \| Parliament \| \| 12 \| Safeway \| \| 13 \| Salem \| \| 14 \| Vogue \| \| 15 \| Wenpor \| \| 16 \| West \| \| 17 \| Winston \| \| 18 \| Air Bar \| \| 19 \| Ismod Nano \| \| 20 \| Nuso \| \| 21 \| Vuse \| \| 22 \| Relx \| \| 23 \| JUUL \| \| 24 \| MAZAJ \| \| 25 \| Puff Bar \| \| 26 \| MarkTen \| \| 27 \| Blu and Logic \| \| 28 \| SMOK \| \| 29 \| Other \| \| 30 \| I prefer not to mention \| | |
| How soon after you wake up do you smoke your first cigarette? | radio   \| 1 \| Within 5 minutes \| \| --- \| --- \| \| 2 \| 6-30 minutes \| \| 3 \| 31-60 minutes \| \| 4 \| After 60 minutes \| | |
| Do you find it difficult to abstain from smoking in places where smoking is prohibited, for example, in the library, in the cinema, airplanes, etc.? | radio   \| 1 \| Yes \| \| --- \| --- \| \| 2 \| No \| | |
| Is smoking more in the first hours after waking up than smoking at any other time of the day? | radio   \| 1 \| Yes \| \| --- \| --- \| \| 2 \| No \| | |
| When is the most difficult time to give up smoking ? | radio   \| 1 \| The Morning \| \| --- \| --- \| \| 2 \| Rest of the day \| | |
| Do you smoke if you are sick and bedridden? | radio   \| 1 \| Yes \| \| --- \| --- \| \| 2 \| No \| | |
| How many do you smoke per day? | radio   \| 1 \| Less than 10 \| \| --- \| --- \| \| 2 \| 11 - 20 \| \| 3 \| 21 - 30 \| \| 4 \| More than 31 \| | |
| On the days when you can smoke both types freely, when do you smoke the first of the day? | radio   \| 1 \| 0-5 mins \| \| --- \| --- \| \| 2 \| 6-15 \| \| 3 \| 16-30 \| \| 4 \| 31-60 \| \| 5 \| 61-120 \| \| 6 \| 121+ \| | |
| Do you sometimes get up at night to smoke? | radio   \| 1 \| Yes \| \| --- \| --- \| \| 2 \| No \| | |
| If yes, how many nights per week do you usually wake up to smoke? | radio   \| 1 \| One night \| \| --- \| --- \| \| 2 \| 2-3 nights \| \| 3 \| More than 3 nights \| | |
| Do you smoke now because it is difficult to quit? (can't stop) | radio   \| 1 \| Yes \| \| --- \| --- \| \| 2 \| No \| | |
| Do you have a strong desire to smoke ? | radio   \| 1 \| Yes \| \| --- \| --- \| \| 2 \| No \| | |
| Over the past week, how strongly did you feel the urge to smoke ? | radio   \| 1 \| None/Slight \| \| --- \| --- \| \| 2 \| Moderate/Strong \| \| 3 \| Very Strong/Extremely Strong \| | |
| Is it difficult not to smoke in non-smoking areas? | radio   \| 1 \| Yes \| \| --- \| --- \| \| 2 \| No \| | |
| When you haven't used for a while or when you're trying to stop using it, do you have tantrums because of your resistance to smoking? | radio   \| 1 \| Yes \| \| --- \| --- \| \| 2 \| No \| | |
| Do you feel stressed or anxious when not smoking ? | radio   \| 1 \| Yes \| \| --- \| --- \| \| 2 \| No \| | |
| What kind of Hookah/Shisha? | checkbox   \| 1 \| AlFakher \| \| --- \| --- \| \| 2 \| Al Nakhla \| \| 3 \| Prefer not to mention \|   Custom alignment: LV | |
| How soon after you wake up do you smoke your first cigarette? | radio   \| 1 \| Within 5 minutes \| \| --- \| --- \| \| 2 \| 6-30 minutes \| \| 3 \| 31-60 minutes \| \| 4 \| After 60 minutes \| \| 5 \| other \| | |
| What is the time to smoke the first Hookah/Shisha after you wake up? | radio   \| 1 \| Morning \| \| --- \| --- \| \| 2 \| Afternoon \| \| 3 \| Night \| | |
| Do you find it difficult to abstain from smoking Hookah/Shisha in places where smoking is prohibited, for example, in the library, in the cinema, airplanes, etc.? | radio   \| 1 \| Yes \| \| --- \| --- \| \| 2 \| No \| | |
| When is the most difficult time to give up smoking Hookah/Shisha | radio   \| 1 \| The Morning \| \| --- \| --- \| \| 2 \| Rest of the day \| | |
| Is smoking Hookah/Shisha more in the first hours after waking up than smoking at any other time of the day? | radio   \| 1 \| Yes \| \| --- \| --- \| \| 2 \| No \| | |
| How many Hookah/Shisha do you smoke per day? | radio   \| 1 \| Less than 1 \| \| --- \| --- \| \| 2 \| 1-2 \| \| 3 \| 2 - 3 \| \| 4 \| More than 3 \| | |
| Do you smoke Hookah/Shisha if you are sick and bedridden? | radio   \| 1 \| Yes \| \| --- \| --- \| \| 2 \| No \| | |
| On the days when you can smoke Hookah/Shisha freely, when do you smoke the first Hookah/Shisha of the day? | radio   \| 1 \| 0-5 mins \| \| --- \| --- \| \| 2 \| 6-15 \| \| 3 \| 16-30 \| \| 4 \| 31-60 \| \| 5 \| 61-120 \| \| 6 \| 121+ \| | |
| Do you sometimes get up at night to smoke? | radio   \| 1 \| Yes \| \| --- \| --- \| \| 2 \| No \| | |
| If yes, how many nights per week do you usually wake up to smoke? | radio   \| 1 \| One night \| \| --- \| --- \| \| 2 \| 2-3 nights \| \| 3 \| More than 3 nights \| | |
| Do you smoke now because it is difficult to quit? (can't stop) | radio   \| 1 \| Yes \| \| --- \| --- \| \| 2 \| No \| | |
| Over the past week, how strongly did you feel the urge to smoke Hookah/Shisha ? | radio   \| 1 \| None/Slight \| \| --- \| --- \| \| 2 \| Moderate/Strong \| \| 3 \| Very Strong/Extremely Strong \| | |
| Do you have a strong desire to smoke Hookah/Shisha? | radio   \| 1 \| Yes \| \| --- \| --- \| \| 2 \| No \| | |
| Is it difficult not to smoke Hookah/Shisha in non-smoking areas? | radio   \| 1 \| Yes \| \| --- \| --- \| \| 2 \| No \| | |
| Do you feel stressed or anxious when not smoking Hookah/Shisha ? | radio   \| 1 \| Yes \| \| --- \| --- \| \| 2 \| No \| | |
| When you haven't used Hookah/Shisha for a while or when you're trying to stop using it, do you have tantrums because of your resistance to smoking? | radio   \| 1 \| Yes \| \| --- \| --- \| \| 2 \| No \| | |
| Section Header: *End of the survey*  If you would like to be nominated for the Research Project Award/incentive, please email us | text | |
| How Did You Hear About this Survey? | radio   \| 1 \| Google \| \| --- \| --- \| \| 2 \| Email \| \| 3 \| WhatsApp \| \| 4 \| Social Media (Twitter, Facebook, Instagram..) \| \| 5 \| Linkedin \| \| 6 \| Referral (add his/her email) \| \| 7 \| Other \| | |
| Email | text | |
| Comments/Suggestions | text | |
